# Unveiling Specificity, Redundancy, and Promiscuity of Five *Saccharomyces cerevisiae* Mitochondrial Carriers

**DOI:** 10.3390/ijms27031450

**Published:** 2026-01-31

**Authors:** Pawel Lojko, Lyubomir Dimitrov Stanchev, Felicia Cara Schulz, Christoph Crocoll, Carlos G. Acevedo-Rocha, Irina Borodina

**Affiliations:** 1 The Novo Nordisk Foundation Center for Biosustainability, Technical University of Denmark, 2800 Kongens Lyngby, Denmark; paloj@biosustain.dtu.dk (P.L.); lydist@biosustain.dtu.dk (L.D.S.);; 2 Section for Molecular Plant Biology, Department of Plant and Environmental Sciences, University of Copenhagen, 1871 Frederiksberg C, Denmark

**Keywords:** transport proteins, *Saccharomyces cerevisiae*, organic acids, amino acids, mitochondria, *Xenopus laevis* oocyte

## Abstract

The transport of metabolites across biological membranes is vital for normal cellular functions, including nutrient uptake, homeostasis, and toxin efflux. In eukaryotes, mitochondrial transporters in the inner mitochondrial membrane (IMM) play a pivotal role in energy production, metabolism, and the biosynthesis of a wide range of compounds. While functional assignments exist for over half of the mitochondrial transporters, emerging high-throughput methodologies underscore the need for reassessment and expansion of the current knowledge, particularly as evidence suggesting functional redundancy and substrate promiscuity has emerged. In this study, we investigated the substrate specificity of five yeast mitochondrial transporters—Crc1 (YOR100c), Ctp1 (YBR291c), Oac1 (YKL120w), Pet9 (YBL030c), and Yhm2 (YMR241w)—via heterologous gene expression in *Xenopus laevis* oocytes and liquid chromatography-mass spectrometry (LC-MS)-based transport assays. We used two substrate mixtures: a 17-compound organic acid mix and a ^13^C-labeled yeast metabolite extract. Our results revealed broader substrate specificities than previously reported, as partially supported by substrate docking simulations. Pet9 transported several organic acids and amino acids, while Yhm2 showed uptake of nine amino acids and fumaric acid. Additional promiscuous transport activity was observed for Crc1, indicating that these proteins may have more extensive metabolic roles than previously known. This study advances the understanding of yeast mitochondrial transporter function, demonstrating redundancy and broad substrate specificity among mitochondrial carriers. It highlights the importance of utilizing in vivo heterologous systems and physiologically relevant substrate mixtures to elucidate transporter functionality.

## 1. Introduction

The transport of metabolites across biological membranes is essential for all living organisms. Metabolite transport ensures key cellular functions such as efficient nutrient import, maintenance of cellular homeostasis, and efflux of toxic compounds. Among other applications, the model organism *Saccharomyces cerevisiae* has been extensively used to understand the functions and molecular mechanisms of membrane proteins involved in metabolite transport. Despite these efforts, roughly two-thirds of the approximately 340 transport proteins in *S. cerevisiae* remain uncharacterized. This is especially the case for transport proteins embedded in the membranes of intracellular compartments, such as vacuoles, peroxisomes, the endoplasmic reticulum (ER), and mitochondria.

Mitochondria, essential organelles in most eukaryotic cells, play a key and fundamental role in energy generation. They produce ATP through the tricarboxylic acid (TCA) cycle and oxidative phosphorylation, both of which are essential for vital cellular functions. Organic acids (OAs) are central in energy generation through ATP [1]. The significance of mitochondria extends beyond energy generation; OAs play a pivotal role in the redox balance [2,3]. Additionally, mitochondria contribute to the metabolism and biosynthesis of amino acids (AAs) such as alanine, glycine, and arginine [4,5,6], as well as various lipids [7], and in the synthesis of iron–sulfur clusters [8], underscoring their vital function in cellular metabolism and biosynthetic pathways.

Unlike other organelles, which are surrounded by a single membrane, mitochondria are enclosed by two very distinct membranes: an outer mitochondrial membrane (OMM) and an inner mitochondrial membrane (IMM). These two membranes are separated by the intermembrane space, a region where protons are pumped during electron transport to create a gradient necessary for ATP synthesis on the IMM [1]. These two membranes serve distinct functions. On one side, the OMM contains porins whose purpose is to facilitate the diffusion of large molecules, ions, and peptides [9]. On the other side is its highly selective counterpart, the IMM. Its purpose is to facilitate the transport of specific metabolites by necessitating the action of mitochondrial carriers (MCs), as well as to take part in the electron transport chain [1]. Beyond metabolite transport, the functional specialization of mitochondrial membranes is also critical for processes such as mitochondrial quality control and turnover, including mitophagy, which has recently been shown to involve SUN family proteins localized to mitochondria [10].

Organic acid (OA) transport between the IMM and the mitochondrial matrix is essential for energy generation and metabolic flux. Although the shuttling of OAs is a vital cellular process, their transport is characterized by redundancy and promiscuity among mitochondrial carriers. Such redundancy confers adaptability to *S. cerevisiae*. It has previously been shown that the deletion of genes encoding specific amino acid (AA) carriers did not affect the viability of *S. cerevisiae* [11], although it resulted in slower growth.

While the substrate specificity of some of the MCs has been identified through in vitro-based assays [12], their specificity might be broader than previously thought. For instance, the complete deletion of the genes encoding the mitochondrial pyruvate carriers 1, 2, and 3 (Mpc1–3) slows the uptake of pyruvate into mitochondria but does not abolish it completely, indicating the presence of pyruvate uptake into mitochondria by other transporters [13]. This redundancy is also evident in other mitochondrial carriers, such as Crc1, which transports carnitine [14] and potentially pyruvate. A quadruple knockout of *MPC1–3* and *CRC1* genes reduced mitochondrial pyruvate uptake but did not eliminate it [13], nor did it impair viability, suggesting a high degree of redundancy in the yeast mitochondrial transportome.

Similarly, previous reports suggest that the knockout of genes encoding AA transporters does not affect *S. cerevisiae*’s viability [11]. In addition to showcasing the robust adaptability of *S. cerevisiae*, this demonstrates a functional redundancy in the transportome, which is most likely not limited to plasma membrane transporters but may also be extended to mitochondrial transporters.

Taking all of these observations into account, the aim of this study is to investigate the substrate specificity and potential promiscuity of five mitochondrial transporters. The selection of these five transporters was guided by phylogenetic analysis and supported by previous evidence in the literature indicating their involvement in organic acid transport. Using a recently established methodology based on heterologous gene expression in *Xenopus laevis* oocytes in combination with a LC-MS based assay [11], five selected yeast mitochondrial transporters were assayed against a selection of OAs and a ^13^C-labeled metabolite mixture. Docking simulations of all identified substrates were further performed for the five transporters. Our results expand the knowledge on transportome redundancy, shedding light on the promiscuity phenomenon present in the mitochondria.

## 2. Results

### 2.1. Phylogenetic Analysis of Mitochondrial Transporters

**Figure 1:**
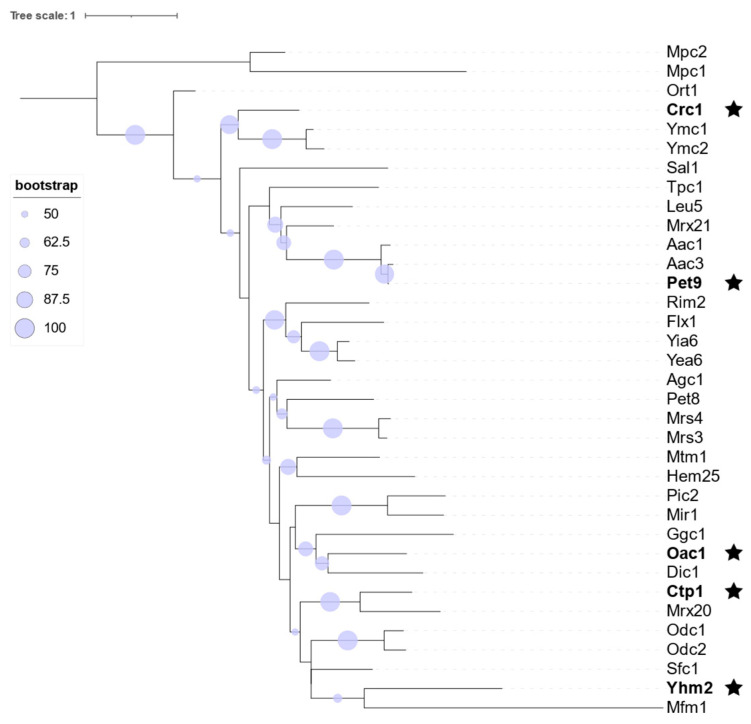
Phylogenetic tree of mitochondrial carriers: Maximum likelihood phylogenetic tree constructed from 35 mitochondrial transporter protein sequences in *S. cerevisiae*. Stars indicate selected transporters in this study. Bootstrap support values are indicated by circle size, with larger circles representing higher support (ranging from 50 to 100). The tree was constructed using IQ-TREE with 285 parsimony-informative sites, and the scale bar indicates one substitution per site.

Phylogenetic inferences of mitochondrial transporters in *S. cerevisiae* showed well-defined clades based on the mitochondrial carrier (MC) family classification (Figure 1). Of the 35 analyzed sequences, 33 fell within the MC family, while Mpc1 and Mpc2 diverged from the main MC clade. Their bootstrap values fell below the threshold of 50, indicating low confidence in their phylogenetic placement. Well-supported clades reflected the high sequence similarity between *S. cerevisiae* mitochondrial transporters, suggesting that they share a common evolutionary origin. However, Mfm1 displayed a markedly longer branch length than the other MC members. Although this could reflect a higher evolutionary rate or long divergence time, the most likely explanation is its low sequence identity (<50%) relative to the other MCs. This can inflate the branch lengths in maximum likelihood reconstructions. Nevertheless, in the overall sequence conservation, functional divergence aligned with phylogenetic structure, indicating that even closely related transporters may have evolved distinct substrate specificities. This supports the hypothesis that sequence identity does not necessarily equate to functional uniformity, and that substrate promiscuity may have arisen within certain evolutionary clades. To test this hypothesis, we selected five transporters—Crc1, Oac1, Ctp1, Yhm2, and Pet9 (Table 1)—to study further promiscuity phenomena by functional assays. The selection of these five transporters was based on both previous evidence for their involvement in OA transport and their positioning in clearly distinct phylogenetic clades. The phylogenetic landscape of mitochondrial transporters in *S. cerevisiae* enables such data-informed decisions, supporting the selection of candidates for transporter functional characterization. The structure and function, combined with phylogenetic analysis, have been utilized previously to compare the relationships between transporter families [15].

The selected transporters were subjected to heterologous gene expression in *X. laevis* oocytes and assayed with two different sets of conditions: (i) an equimolar concentration of a 17-organic-acid mixture (OAM) (Supplementary Table S1), and (ii) a ^13^C-labeled metabolite mixture from *Pichia pastoris* to study potential AA transport.

### 2.2. Transport of Organic Acids by Selected Mitochondrial Transporters

**Figure 2:**
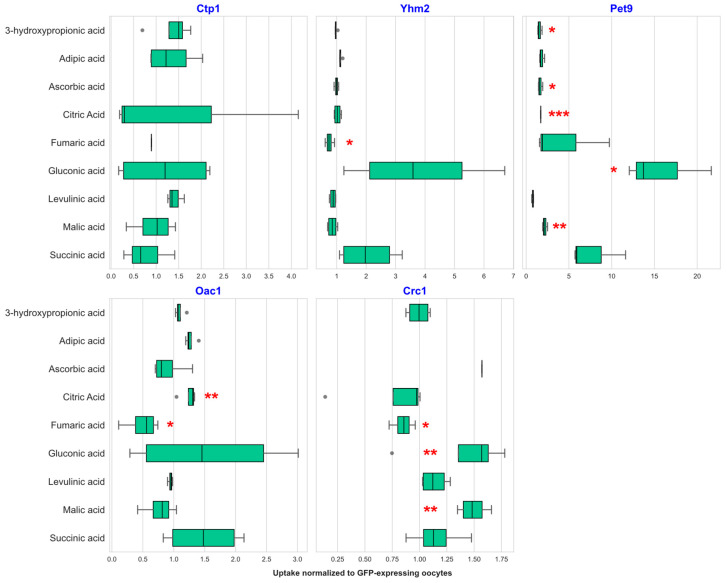
Uptake of organic acids into *X. laevis* oocytes: Boxplot of the uptake of nine different OAs into *X. laevis* oocytes heterologously producing five different mitochondrial transporters. Boxplots represent the peak area of uptake of each individual organic acid (*x*-axis) into transporter-producing oocytes normalized to the mean of the peak area for uptake into GFP-producing control oocytes. Four replicates were analyzed per transporter. Boxplot: The box boundary represents the interquartile range (IQR), while the whiskers represent the 1.5× maximum and minimum values, gray dots depict outliers, and the line in the box represents the 50% median value. Statistical significance was evaluated by the Welch *t*-test (ANOVA): * < 0.05, ** < 0.01, *** < 0.001.

From the 17-compound OAM, using our LC-MS method, we unequivocally detected ascorbic acid, citric acid, gluconic acid, succinic acid, adipic acid, 3-hydroxypropionic acid (3-HP), levulinic acid, fumaric acid, and malic acid. The remaining OAs (Supplementary Table S1) were not resolved in the LC-MS due to structural similarities and overlapping fragmentation patterns. The largest perturbation of OA uptake profile was observed for Pet9 amongst the analyzed carriers. This transporter showed uptake for multiple OAs (Figure 2). Another transporter that was able to facilitate the uptake of multiple organic acids was Crc1. This MC showed significant uptake of fumaric acid (0.85-fold), gluconic acid (1.42-fold), and malic acid (1.49-fold) compared to the low background uptake of fumaric acid (94.86 ± 11.86 AU), gluconic acid (44.27 ± 20.30 AU), and malic acid (7851 ± 1522 AU) in the control GFP-producing oocytes (Figure 2). Oac1 showed significant uptake for fumaric acid (0.49-fold) and citric acid (1.24-fold) (Figure 2). Yhm2 displayed significant uptake for only one compound, fumaric acid (0.74-fold) (Figure 2). Lastly, Ctp1 was the only transporter that did not show any significant uptake for any of the detectable OAs in the tested mixture.

### 2.3. Transport of Amino Acids by Selected Yeast Mitochondrial Transporters

**Figure 3:**
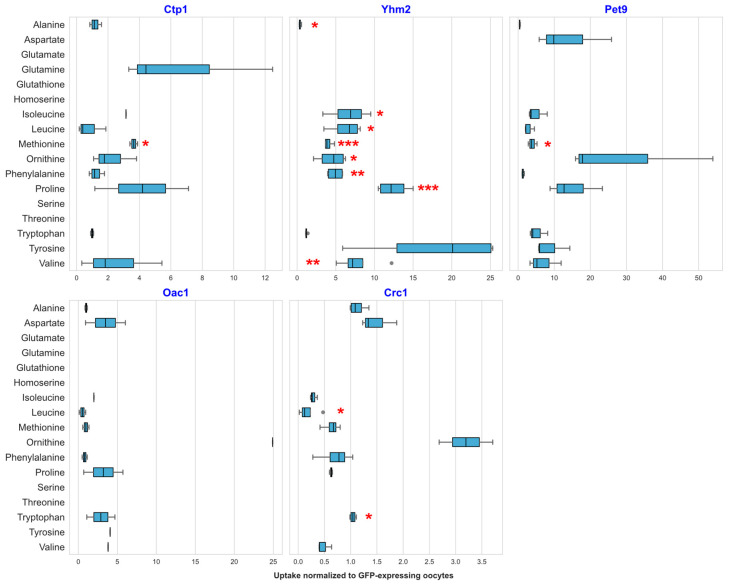
Uptake of ^13^C-labeled amino acids into *X. laevis* oocytes: Boxplot of the uptake of 17 AAs into *X. laevis* oocytes heterologously producing five different mitochondrial transporters. Data shown represent the peak area of uptake of each individual amino acid (*x*-axis) into transporter-producing oocytes. Each replicate, consisting of 3–6 individual oocytes, was normalized to the mean of the control (heterologously expressing the GFP gene), which was a biological quadruplicate consisting of 3–6 oocytes. Four replicates were used per transporter. Boxplot: The box boundary represents the interquartile range (IQR), while the whiskers represent the 1.5× maximum and minimum values, gray dots depict outliers, and the line in the box represents the 50% median value. Statistical significance was evaluated by the Welch *t*-test (ANOVA): * < 0.05, ** < 0.01, *** < 0.001.

To determine whether the selected transporters garner greater promiscuity towards different types of metabolites, we further investigated the chosen transporters against the ^13^C-labeled *P. pastoris* metabolite extract. This yeast metabolite extract, with a large variety of metabolites in physiological concentrations, mimics the native yeast environment. Transporter proteins, facing the extracellular milieu, are thus exposed to their potential substrates in a setting more closely resembling their native environment. We expressed the genes encoding for the transporters in *X. laevis* oocytes and carried out the uptake assay in the same way as previously mentioned, but using ^13^C-labeled *P. pastoris* metabolite extract instead of the OAM, and using a different LC-MS methodology. Yhm2 transported the largest number of ^13^C-labeleled AAs (Figure 3). Significant uptake was observed for alanine (0.34-fold), isoleucine (6.67-fold), leucine (6.29-fold), ornithine (4.46-fold), phenylalanine (4.93-fold), valine (7.91-fold), proline (12.5-fold), and methionine (4.05-fold). Aspartate was likewise observed to be imported into the oocytes producing Yhm2 but not into the control oocytes, which only produced cytosolic GFP (Supplementary Figure S1A). Compared with no uptake into control GFP-injected oocytes, tryptophan and leucine were observed to be transported by Crc1-producing oocytes; and glutamate, glutathione, and threonine were observed to be transported by Pet9-producing oocytes with no uptake into control oocytes (Supplementary Figure S1B). Interestingly, methionine (3.91-fold) was likewise taken up by Pet9-producing oocytes in a significant manner compared with the control group of oocytes (48.14 ± 16.10 AU) (Figure 3). Ctp1 likewise showed significant uptake for methionine (3.64-fold) compared to the GFP-producing controls, as well as for aspartate, where none was present in the controls (Supplementary Figure S1A).

In addition, molecular docking was used to assess transporter–substrate binding and compare predictions with in vivo transport data, using reported substrates as controls and AlphaFold structures where needed. Strong binding was correctly predicted for the known Pet9 substrates ADP and ATP, while most other interactions were weaker, with several moderate-affinity interactions matching experimental uptake and others not observed experimentally (Supplementary Figure S2).

### 2.4. Hierarchical Cluster Analysis for Uptake of OAs and AAs

Similar substrate specificities might indicate overlapping or redundant transporter functions. In order to investigate the differences and similarities among the five transporters based on the identified substrates, we next conducted hierarchical cluster analysis for OAs and AAs.

**Figure 4:**
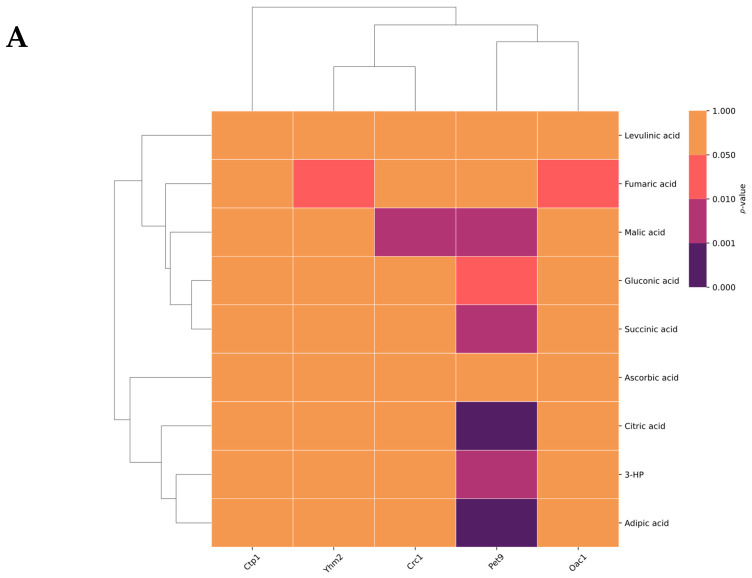

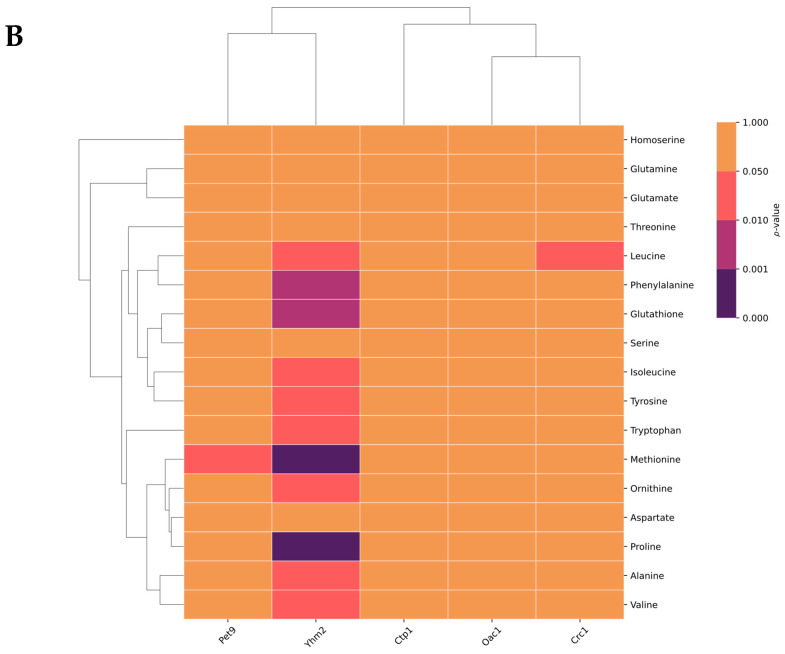
Hierarchical cluster analysis of uptake of OAs (**A**) and AAs (**B**) by mitochondrial transporters produced in *X. laevis* oocytes. The heatmap depicts the statistical significance (*p*-values) of the uptake of either OAs, consisting of a mixture, or AAs from a ^13^C-labeled metabolite mixture. The significance of the uptake is represented by the color intensity, ranging from salmon (<0.05) to purple (<0.01) and dark purple (<0.001). The dendrograms on the left and top sides cluster the OAs/AAs and transporters based on similarity. The hierarchical clustering and heatmap visualization were performed using Seaborn’s cluster function, which utilizes Ward’s linkage method with Euclidean distance.

The transporters Oac1 and Pet9 formed a distinct cluster suggesting commonalities between either their function or substrate specificity (Figure 4). A second cluster emerged from the transporters Yhm2 and Crc1, whose involvement with the transport of OAs fell within a similar pattern. Unsurprisingly, Ctp1 displayed no meaningful similarities with the other transporters, as it did not facilitate the uptake of any of the tested OAs. The OAs clustered together into two distinct groups: one defined by malic acid and succinic acid, and another formed by citric acid, 3-hydroxypropionic acid, and adipic acid. The remaining compounds—ascorbic acid, levulinic acid, fumaric acid, and gluconic acid—did not form any distinct clusters.

Exploration of the relationship between the selected transporters and *P. pastoris* metabolite extract revealed a distinct clustering pattern associated with the metabolites and transporters. Yhm2 stood out, as it exhibited a distinct pattern of metabolite interactions and transporter specificity of the metabolites, whereas methionine, phenylalanine, and glutathione displayed the most significant transporter-associated interactions. Conversely, leucine and ornithine had a lower degree of transporter-associated interactions. Notably, Pet9 and Yhm2 clustered together, suggesting a close relationship, while the remaining transporters (Ctp1, Oac1, and Crc1) formed a distinct separate cluster. Several of the screened AAs clustered into distinct groups; notably, the amino acids methionine, ornithine, and phenylalanine and the tripeptide glutathione formed a cluster distinct from the rest of the screened AAs, pertaining to significant interactions with the transporters.

## 3. Discussion

In this study, we functionally re-evaluated five *S. cerevisiae* MCs by heterologously producing them in *X. laevis* oocytes and screening them against two complex substrate mixtures comprising of a set of 17 OAs and a ^13^C-labeled yeast metabolite extract. In contrast to previous studies, which typically assessed individual substrates using reconstituted proteoliposome assays, our in vivo system enabled the detection of a broad range of transport events under near-physiological membrane conditions with minimal background transport activity. This methodology revealed a substantially wider substrate repertoire for each of the examined transporters than has previously been reported.

Among our five selected carriers, Yhm2 exhibited the most pronounced functional expansion, showing significant transport not only for OAs but also for several AAs—particularly methionine and proline—as well as the tripeptide glutathione. This is the first in vivo evidence of AA transport by the Yhm2 transporter, which suggests a dual-purpose role of the transporter in both OA and AA metabolism. This expansion of functionality suggests far greater implication in the metabolism and transport of AAs than has widely been associated with the aspartate–glutamate carrier Agc1 [20] and the s-adenosylmethionine carrier Pet8 [21], as well as through indirect transport through the oxodicarboxylate carriers Odc1 and Odc2 [22].

Other carriers in our analysis displayed extended transport profiles. Ctp1, the carrier traditionally associated with citrate transport [16], showed unexpected uptake for methionine and aspartate, suggesting possible implications in nitrogen metabolism in addition to its canonical OA transport. Oac1, a known oxaloacetate/sulfate transporter [18], transported fumarate and citrate in our assay, while Crc1, conventionally linked to carnitine shuttling, imported several AAs and OAs when assayed under in vivo conditions. Pet9, the essential ADP/ATP carrier, also transported several non-canonical substrates, including the OAs malic acid, gluconic acid, succinic acid, citric acid, adipic acid, and 3-hp, as well as the AA methionine and the tripeptide glutathione. Although Pet9’s primary role remains adenine nucleotide exchange, it has also been implicated in the transport of porphyrin derivatives [23]. Such results support emerging evidence that adenine nucleotide carriers may possess broader substrate capacities depending on structural context and membrane composition. The human paralog ANT1, which shares 73% sequence identity with PET9, has been implicated in the transport of charged fatty acids via a “Fatty Acid sliding mechanism” [24]. This mechanism could potentially explain the transport of anionic OAs observed in our study, given their classification as short-chain fatty acids.

Another mechanism that has gained traction is how the lipid composition affects the transporter activity [25]. It has been shown that lipid composition (mainly cholesterol) has an influence on the gating effect of ion channels [26]; it has likewise been shown that altering the cholesterol and sphingolipid contents alters the selectivity of aquaporins towards water permeability [27]. More evidence has also been seen for the anionic lipids’ ability to alter the channel activity of certain ion channels [28]; the proximity of the anionic lipids to certain salt bridges within the channels would positively modulate the channel [28]. For MCs such as Pet9, it has recently been demonstrated how cardiolipin modulates MC transport activity [29]. With these recent findings, it is possible that the substrate preference of our chosen MCs is modulated when embedded in the *Xenopus* plasma membrane, the composition of which is slightly different from the yeast mitochondrial IMM. Primarily, it consists of phosphatidylcholine (PC) and sphingomyelin (SM), accounting for 62% of the total phospholipid content, while phosphatidylethanolamine (PE) sits at 22%, phosphatidylinositol (PI) at 7%, and phosphatidylserine (PS) at 5% [30]. Cholesterol is likewise present but measured in a different manner. The mitochondrial IMM, on the other hand, consists of 38% PC, 24% PE, 16% PI, 4% PS, 2% phosphatidic acid, and 16% cardiolipin [31], while the OMM contains 46% PC, 33% PE, 10% PI, 1% PS, 4% phosphatic acid, and finally 6% cardiolipin. As stated previously, the heterologous production of our MCs in the *X. laevis* oocyte plasma membrane—where more cholesterol and sphingomyelin lipids, along with low amounts of anionic lipids, are found—likely modulates the MCs in a different manner, allowing for the uptake of a wide range of substrates. Nevertheless, our results point towards a broader substrate profile of the MCs than was previously established.

Importantly, the expanded transport activities observed in this study have direct implications for central mitochondrial metabolism. Many of our identified substrates, such as fumarate, citrate, and malate, are key intermediates of the TCA cycle. Transport of these metabolites can therefore influence oxidative metabolism, biosynthetic reactions, and redox balancing. In this context, the increased promiscuity of the MCs provides alternative entry and exit points for these metabolites, which, in turn, contribute to the metabolic flexibility under varying physiological conditions.

In addition, the observed transport of AAs and related metabolites, such as methionine, proline, and glutathione, links MC activity to AA biosynthesis, nitrogen metabolism, and cellular redox homeostasis. Altered transport capacity of the MCs may therefore modulate the exchange of metabolites between the mitochondrial matrix and cytosol, supporting a broader and more robust energy balance and biosynthetic capacity, contributing to more adaptive transport capacity of mitochondria.

Molecular docking was used to evaluate whether computational predictions could support the experimentally observed broad substrate specificity, despite known limitations for transporters due to their dynamic conformations [32,33]. The analysis identified several moderate-affinity interactions that aligned with experimental transport activity but also showed discrepancies likely arising from the high structural similarity of AlphaFold-predicted transporter models (Supplementary Figures S2 and S3, Supplementary Tables S4 and S5). Nonetheless, docking controls for the crystallized transporter Pet9 (YBL030c) showed expected binding to known substrates and supported several experimentally observed interactions, providing partial validation of the experimental results.

Altogether, the discrepancies in substrate specificities for the five selected MCs in this study compared to previous works might be due to the use of different methodologies. First, we employed *Xenopus* oocytes as an in vivo system for studying *S. cerevisiae* mitochondrial transporters, while previous studies have utilized in vitro proteoliposome assays. Both systems provide a platform for single-transporter characterization. However, the *Xenopus* plasma membrane can be dynamically modulated by the cell and has a well-characterized lipid composition, whereas the proteoliposomal membrane constitutes a static lipid environment, which can be designed in the pre-experimental phase but does not constitute a dynamic or native environment. Second, the differences in the number of substrates as well as their concentrations, combined with a different method of detection used in our study compared to previous works, could also have given rise to the broader observed substrate specificities of the five selected transporters.

## 4. Materials and Methods

### 4.1. Materials

Cloning materials were obtained from New England Biolabs (Ipswich, MA, USA) or Thermo Scientific (Waltham, MA, USA). Gene sequencing services were provided by Eurofins Genomics (Ebersberg, Germany), and oligonucleotides were purchased from Integrated DNA Technologies (Leuven, Belgium). Yeast metabolite extract from *Pichia pastoris* U-^13^C (98%) (^13^C-PP) was purchased from Euriso-Top (Saarbrücken, Germany). All other materials were purchased from Sigma Aldrich (München, Germany), unless stated otherwise.

### 4.2. Cloning and Plasmid Construction

The *E. coli* DH5α strain was used for plasmid amplification, and successful transformants were selected on LB plates with 100 µg/mL ampicillin. Cloning of transporter genes for cRNA expression in *X. laevis* oocytes was completed as previously described [34]. Briefly, mitochondrial transporter genes were first cloned into pCfB5245 plasmids encoding for T7p-β-globin 5-UTR and β-globin 3-UTR [35]. The resulting plasmids were then used as a PCR template for the amplification of the cassette fragment for in vitro RNA synthesis. Capped RNAs for transporter production in oocyte cells were then synthesized with the T7 mMESSAGE mMACHINE™ kit (Thermo Fisher Scientific, Waltham, MA, USA). The quality of the cRNAs (>500 ng/µL) was analyzed with the Agilent 2100 Bioanalyzer from Agilent Technologies (Waldbronn, Germany) before proceeding with oocyte injection. All oligonucleotides and plasmids are listed in Supplementary Tables S2 and S3, respectively.

### 4.3. Transport Assay in Oocyte Cells

Defolliculated *X. laevis* oocytes, stages V–VI, were purchased from EcoCyte Bioscience GmbH (Dortmund, Germany). Oocytes were injected with 50 nL of cRNA (400 ng/µL transporter cRNA and 100 ng/µL GFP cRNA) using an automated RoboInject device (Multichannel System, Reutlingen, Germany) and incubated for three days at 18 °C. Afterwards, GFP-positive cells were selected and used further for the uptake assay.

Three to six oocyte cells per biological replicate were pre-incubated in a Kulori buffer (90 mM NaCl, 1 mM KCl, 1 mM MgCl_2_, 1 mM CaCl_2_, 5 mM HEPES, pH 7.4) supplemented with gentamycin (100 μg/mL) and then transferred to ^13^C-PP at pH 7.4 or OAM at pH 5.0 (Supplementary Table S1) for 1 h at room temperature. Cells were subsequently transferred to 4 °C Kulori buffer to stop the assay and washed three times in fresh Kulori buffer before being disrupted with 100 µL of ice-cold 50% (*v*/*v*) methanol. Cell lysates were incubated for 2 h at −20 °C and then spun down to remove cell debris. Finally, 70 µL from the supernatant was mixed with 50 µL of water before subjecting it to LC-MS analysis.

### 4.4. Liquid Chromatography-Mass Spectrometry

OAs and yeast metabolite extracts in the diluted oocyte extracts were subjected to analysis by LC-MS. Briefly, chromatography was performed on a 1290 Infinity II UHPLC system (Agilent Technologies). Separation was achieved on a Zorbax RRHD Eclipse XDB-C18 column (150 × 3.0 mm, 1.8 µm, Agilent Technologies). Formic acid in water (0.05%, *v*/*v*) and acetonitrile (supplied with 0.05% formic acid, *v*/*v*) were employed as mobile phases A and B, respectively. The elution profile was as follows: 0.0–0.5 min, 3% B; 0.5–5.95 min, 3–30% B; 5.95–6.0 min 30–98% B; 6.0–7.0 min, 98% B; 7.0–7.1 min, 98–3% B; and 7.1–8.0 min, 3% B. The mobile-phase flow rate was 400 µL/min. The column temperature was maintained at 40 °C. The LC was coupled to an Ultivo triple-quadrupole MS (Agilent Technologies) equipped with a Jetstream electrospray ionization (ESI) source. The ion spray voltage was set to +4000 V and 3500 in positive and negative ion modes, respectively. The dry gas temperature was set to 325 °C, and the dry gas flow was set to 10 L/min. The sheath gas temperature was set to 250 °C and the sheath gas flow to 12 L/min. The nebulizing gas was set to 40 psi. Nitrogen was used as the dry gas, nebulizing gas, and collision gas. The instrument parameters were optimized for the best detection of all metabolites with mixes of reference standards. Multiple reaction monitoring (MRM) was used to monitor precursor ion → fragment ion transitions. Both the Q1 and Q3 quadrupoles were maintained at unit resolution. Mass Hunter Quantitation Analysis for QQQ software (Version 10.1, Agilent Technologies) was used for data processing.

### 4.5. Data Analysis

Raw data from LC-MS was analyzed and processed with Mass Hunter Quantitation Analysis for QQQ software (Version 10.1, Agilent Technologies). Standard settings were used for peak integration (Integrator: Agile2, Noise algorithm: RMS, noise SD multiplier: 5.0). Statistical analyses and figure generation were performed using Python (version 2.6.3) and the following packages: NumPy (version 1.26.4) [36], Pandas (version 2.2.1) [37,38], SciPy (version 1.13.0) [39], matplotlib (version 3.8.0) [40], and Seaborn (version 0.13.1) [41]. All scripts were run in Jupyter Notebooks (version 7.0.3).

### 4.6. Phylogenetic Tree Construction

A total of 35 amino acid sequences were manually downloaded from the Uniprot database [42] in .fasta format, and multiple sequence alignment was performed with Muscle5 [43]. Subsequently, trimAl v2.0 was used for automated alignment trimming [44]. Phylogenomic inferences were constructed with IQ-Tree v2.4 [45,46] using 285 parsimony-informative sites, 644 singleton sites, and 1078 constant sites. An initial parsimony tree was performed using the LG + I + G model and maximum likelihood to choose the best-fitting model with ModelFinder [47], which resulted in LG + F + I + G4 after 20 iterations. By applying Ultrafast Bootstrap (UFBoot) [48,49], the bootstrap correlation coefficient of split occurrence frequencies was 0.995 after 300 iterations. Finally, a consensus tree was produced and exported. The tree visualizations and annotations were generated using iTOL v6 [50]. All packages were downloaded from Github via Bioconda [51] and were used in conjunction with Python.

### 4.7. Molecular Docking Analysis

Molecular docking analysis was performed for five of the transporters with all AAs and OAs detected in the assay mixtures. The structures of all transporters used for docking were obtained from the AlphaFold Protein Structure Database [52,53]. The 3D substrate structures were retrieved from PubChem [54] and ChEBI [55]. The protein and ligand input files were prepared and loaded using Meeko (version 0.6.1 Forli Lab, 2024), OpenMM (version 8.2.0) [56], and RDKit (version 2023.09.6) [57]. Structure superimposition was performed using PyMOL (the PyMOL Molecular Graphics System, Version 3.1.3 Schrödinger, LLC, NY, USA).

The molecular docking simulations were carried out with AutoDock Vina (version 1.2.7) [58,59], with the exhaustiveness parameter set to 32. The search space for docking was defined by grid boxes measuring 30 × 30 × 30 Å^3^, which were placed around the centroids of the proteins. Minor adjustments were made to center the grids on the expected binding cavities, which were modeled after the region identified as the substrate-binding site in [21]. The precise box specifications are listed in Supplementary Table S5.

The top ten scoring poses were further investigated using PyMOL and UCSF Chimera (version 1.10rc202505210000) [60], and atom-level interactions were identified with PLIP 2025 [61] for visualization. Finally, the top scoring positions for each protein–ligand interaction were further analyzed and visualized in a heatmap using Matplotlib (version 3.10.1) [41], Pandas (version 2.1.4) [38], and Seaborn (version 0.13.2) [41].

## 5. Conclusions

Our study reveals previously overlooked redundancies and suggests new substrate specificities for five mitochondrial transporters in *S. cerevisiae*. In addition to investigating the functional overlap in the mitochondrial transport systems, this work expands on the existing knowledge about the substrate specificity and range of the selected MCs. By mimicking yeast’s native environment, with a rich availability of OAs and AAs, we propose Yhm2 to be involved in the transport of eleven AAs and fumaric acid. Furthermore, our findings indicate that Pet9 has a broader substrate specificity than previously thought, being able to facilitate the transport of six OAs. This study expands our understanding about mitochondrial transporter redundancy, which could potentially guide future efforts in metabolic engineering strategies that rely on the engineering of transporters for the production of valuable compounds.

## Figures and Tables

**Table 1:** Mitochondrial transporters analyzed in this study.

Transporter	Gene	Reported Substrate(s)	Reference
Crc1	*YOR100c*	Carnitine *	[14]
Ctp1	*YBR291c*	Citrate *	[16]
Oac1	*YKL120w*	2-Isopropylmalate *, oxaloacetate *, sulfate	[17,18]
Pet9	*YBL030c*	ADP */ATP	[19]
Yhm2	*YMR241w*	Citrate *, fumarate *, oxoglutarate *	[12]

* Transport determined through radio-labeled assays.

## Data Availability

The original contributions presented in this study are included in the article/Supplementary Materials. Further inquiries can be directed to the corresponding author.

## Supplementary Materials

The following supporting information can be downloaded at: https://www.mdpi.com/article/10.3390/ijms27031450/s1.

## Abbreviations

AA	Amino acid
*E. coli*	*Escherichia coli*
GFP	Green fluorescent protein
IMM	Inner mitochondrial membrane
LC-MS	Liquid chromatography-mass spectrometry
MC	Mitochondrial carrier
MPC	Mitochondrial pyruvate carrier
OA	Organic acid
OAM	Organic acid mix
OMM	Outer mitochondrial membrane
*S. cerevisiae*	*Saccharomyces cerevisiae*
TCA cycle	Tricarboxylic acid cycle
*X. laevis*	*Xenopus laevis*

## References

[1] Nelson D.L., Cox M.M. (2021). Lehninger Principles of Biochemistry.

[2] Martínez-Reyes I., Chandel N.S. (2020). Mitochondrial TCA Cycle Metabolites Control Physiology and Disease. Nat. Commun..

[3] Nunnari J., Suomalainen A. (2012). Mitochondria: In Sickness and in Health. Cell.

[4] Maaheimo H., Fiaux J., Çakar Z.P., Bailey J.E., Sauer U., Szyperski T. (2001). Central Carbon Metabolism of *Saccharomyces cerevisiae* Explored by Biosynthetic Fractional ^13^C Labeling of Common Amino Acids. Eur. J. Biochem..

[5] Ljungdahl P.O., Daignan-Fornier B. (2012). Regulation of Amino Acid, Nucleotide, and Phosphate Metabolism in *Saccharomyces cerevisiae*. Genetics.

[6] Li Q., Hoppe T. (2023). Role of Amino Acid Metabolism in Mitochondrial Homeostasis. Front. Cell Dev. Biol..

[7] Houten S.M., Wanders R.J.A. (2010). A General Introduction to the Biochemistry of Mitochondrial Fatty Acid Β-oxidation. J. Inherit. Metab. Dis..

[8] Braymer J.J., Lill R. (2017). Iron–Sulfur Cluster Biogenesis and Trafficking in Mitochondria. J. Biol. Chem..

[9] Hodge T., Colombini M. (1997). Regulation of Metabolite Flux through Voltage-Gating of VDAC Channels. J. Membr. Biol..

[10] Meng S., Chao S., Xiong M., Cheng L., Sun Y., Wang L., Chen Y., Jane S.J., Luo C., Chen J. (2025). CaSun1, a SUN Family Protein, Governs the Pathogenicity of *Colletotrichum camelliae* by Recruiting CaAtg8 to Promote Mitophagy. Hortic. Res..

[11] Stanchev L.D., Møller-Hansen I., Lojko P., Rocha C., Borodina I. (2023). Screening of *Saccharomyces cerevisiae* Metabolite Transporters by 13C Isotope Substrate Labeling. Front. Microbiol..

[12] Castegna A., Scarcia P., Agrimi G., Palmieri L., Rottensteiner H., Spera I., Germinario L., Palmieri F. (2010). Identification and Functional Characterization of a Novel Mitochondrial Carrier for Citrate and Oxoglutarate in *Saccharomyces cerevisiae*. J. Biol. Chem..

[13] Timón-Gómez A., Proft M., Pascual-Ahuir A. (2013). Differential Regulation of Mitochondrial Pyruvate Carrier Genes Modulates Respiratory Capacity and Stress Tolerance in Yeast. PLoS ONE.

[14] Palmieri L., Lasorsa F.M., Iacobazzi V., Runswick M.J., Palmieri F., Walker J.E. (1999). Identification of the Mitochondrial Carnitine Carrier in *Saccharomyces cerevisiae*. FEBS Lett..

[15] Chen J.S., Reddy V., Chen J.H., Shlykov M.A., Zheng W.H., Cho J., Yen M.R., Saier M.H. (2011). Phylogenetic Characterization of Transport Protein Superfamilies: Superiority of SuperfamilyTree Programs over Those Based on Multiple Alignments. Microb. Physiol..

[16] Kaplan R.S., Mayor J.A., Gremse D.A., Wood D.O. (1995). High Level Expression and Characterization of the Mitochondrial Citrate Transport Protein from the Yeast *Saccharomyces cerevisiae*. J. Biol. Chem..

[17] Palmieri L., Lasorsa F.M., Vozza A., Agrimi G., Fiermonte G., Runswick M.J., Walker J.E., Palmieri F. (2000). Identification and Functions of New Transporters in Yeast Mitochondria. Biochim. Biophys. Acta (BBA)-Bioenerg..

[18] Palmieri L., Vozza A., Agrimi G., De Marco V., Runswick M.J., Palmieri F., Walker J.E. (1999). Identification of the Yeast Mitochondrial Transporter for Oxaloacetate and Sulfate. J. Biol. Chem..

[19] Mavridou V., King M.S., Tavoulari S., Ruprecht J.J., Palmer S.M., Kunji E.R.S. (2022). Substrate Binding in the Mitochondrial ADP/ATP Carrier Is a Step-Wise Process Guiding the Structural Changes in the Transport Cycle. Nat. Commun..

[20] Cavero S., Vozza A., Del Arco A., Palmieri L., Villa A., Blanco E., Runswick M.J., Walker J.E., Cerdán S., Palmieri F. (2003). Identification and Metabolic Role of the Mitochondrial Aspartate-glutamate Transporter in *Saccharomyces cerevisiae*. Mol. Microbiol..

[21] Marobbio C.M.T. (2003). Identification and Functional Reconstitution of Yeast Mitochondrial Carrier for S-Adenosylmethionine. EMBO J..

[22] Palmieri L., Agrimi G., Runswick M.J., Fearnley I.M., Palmieri F., Walker J.E. (2001). Identification in *Saccharomyces cerevisiae* of Two Isoforms of a Novel Mitochondrial Transporter for 2-Oxoadipate and 2-Oxoglutarate. J. Biol. Chem..

[23] Azuma M., Kabe Y., Kuramori C., Kondo M., Yamaguchi Y., Handa H. (2008). Adenine Nucleotide Translocator Transports Haem Precursors into Mitochondria. PLoS ONE.

[24] Kreiter J., Škulj S., Brkljača Z., Bardakji S., Vazdar M., Pohl E.E. (2023). FA Sliding as the Mechanism for the ANT1-Mediated Fatty Acid Anion Transport in Lipid Bilayers. Int. J. Mol. Sci..

[25] Drew D., Boudker O. (2024). Ion and Lipid Orchestration of Secondary Active Transport. Nature.

[26] Jiang Q.-X. (2019). Cholesterol-Dependent Gating Effects on Ion Channels. Cholesterol Modulation of Protein Function.

[27] Tong J., Briggs M.M., McIntosh T.J. (2012). Water Permeability of Aquaporin-4 Channel Depends on Bilayer Composition, Thickness, and Elasticity. Biophys. J..

[28] Schmidpeter P.A.M., Wu D., Rheinberger J., Riegelhaupt P.M., Tang H., Robinson C.V., Nimigean C.M. (2022). Anionic Lipids Unlock the Gates of Select Ion Channels in the Pacemaker Family. Nat. Struct. Mol. Biol..

[29] Senoo N., Chinthapalli D.K., Baile M.G., Golla V.K., Saha B., Oluwole A.O., Ogunbona O.B., Saba J.A., Munteanu T., Valdez Y. (2024). Functional Diversity among Cardiolipin Binding Sites on the Mitochondrial ADP/ATP Carrier. EMBO J..

[30] Hill W.G., Southern N.M., MacIver B., Potter E., Apodaca G., Smith C.P., Zeidel M.L. (2005). Isolation and Characterization of the *Xenopus* Oocyte Plasma Membrane: A New Method for Studying Activity of Water and Solute Transporters. Am. J. Physiol.-Ren. Physiol..

[31] Simbeni R., Pon L., Zinser E., Paltauf F., Daum G. (1991). Mitochondrial Membrane Contact Sites of Yeast. Characterization of Lipid Components and Possible Involvement in Intramitochondrial Translocation of Phospholipids. J. Biol. Chem..

[32] Zhang Y., Newstead S., Sarkies P. (2025). Predicting Substrates for Orphan Solute Carrier Proteins Using Multi-Omics Datasets. BMC Genom..

[33] Kroll A., Niebuhr N., Butler G., Lercher M.J. (2024). SPOT: A Machine Learning Model That Predicts Specific Substrates for Transport Proteins. PLoS Biol..

[34] Wang G., Møller-Hansen I., Babaei M., D’Ambrosio V., Christensen H.B., Darbani B., Jensen M.K., Borodina I. (2021). Transportome-Wide Engineering of *Saccharomyces cerevisiae*. Metab. Eng..

[35] Darbani B., Motawia M.S., Olsen C.E., Nour-Eldin H.H., Møller B.L., Rook F. (2016). The Biosynthetic Gene Cluster for the Cyanogenic Glucoside Dhurrin in Sorghum Bicolor Contains Its Co-Expressed Vacuolar MATE Transporter. Sci. Rep..

[36] Harris C.R., Millman K.J., van der Walt S.J., Gommers R., Virtanen P., Cournapeau D., Wieser E., Taylor J., Berg S., Smith N.J. (2020). Array Programming with NumPy. Nature.

[37] McKinney W. Data Structures for Statistical Computing in Python. Proceedings of the 9th Python in Science Conference.

[38] Pandas Development Team Pandas 2023. https://pandas.pydata.org/docs/whatsnew/v2.2.1.html.

[39] Virtanen P., Gommers R., Oliphant T.E., Haberland M., Reddy T., Cournapeau D., Burovski E., Peterson P., Weckesser W., Bright J. (2020). SciPy 1.0: Fundamental Algorithms for Scientific Computing in Python. Nat. Methods.

[40] Hunter J.D. (2007). Matplotlib: A 2D Graphics Environment. Comput. Sci. Eng..

[41] Waskom M. (2021). Seaborn: Statistical Data Visualization. J. Open Source Softw..

[42] Bateman A., Martin M.-J., Orchard S., Magrane M., Ahmad S., Alpi E., Bowler-Barnett E.H., Britto R., Bye-A-Jee H., Cukura A. (2023). UniProt: The Universal Protein Knowledgebase in 2023. Nucleic Acids Res..

[43] Edgar R.C. (2022). Muscle5: High-Accuracy Alignment Ensembles Enable Unbiased Assessments of Sequence Homology and Phylogeny. Nat. Commun..

[44] Capella-Gutiérrez S., Silla-Martínez J.M., Gabaldón T. (2009). TrimAl: A Tool for Automated Alignment Trimming in Large-Scale Phylogenetic Analyses. Bioinformatics.

[45] Nguyen L.-T., Schmidt H.A., von Haeseler A., Minh B.Q. (2015). IQ-TREE: A Fast and Effective Stochastic Algorithm for Estimating Maximum-Likelihood Phylogenies. Mol. Biol. Evol..

[46] Minh B.Q., Schmidt H.A., Chernomor O., Schrempf D., Woodhams M.D., von Haeseler A., Lanfear R. (2020). IQ-TREE 2: New Models and Efficient Methods for Phylogenetic Inference in the Genomic Era. Mol. Biol. Evol..

[47] Kalyaanamoorthy S., Minh B.Q., Wong T.K.F., von Haeseler A., Jermiin L.S. (2017). ModelFinder: Fast Model Selection for Accurate Phylogenetic Estimates. Nat. Methods.

[48] Minh B.Q., Nguyen M.A.T., von Haeseler A. (2013). Ultrafast Approximation for Phylogenetic Bootstrap. Mol. Biol. Evol..

[49] Hoang D.T., Chernomor O., von Haeseler A., Minh B.Q., Vinh L.S. (2018). UFBoot2: Improving the Ultrafast Bootstrap Approximation. Mol. Biol. Evol..

[50] Letunic I., Bork P. (2024). Interactive Tree of Life (ITOL) v6: Recent Updates to the Phylogenetic Tree Display and Annotation Tool. Nucleic Acids Res..

[51] Grüning B., Dale R., Sjödin A., Chapman B.A., Rowe J., Tomkins-Tinch C.H., Valieris R., Köster J. (2018). Bioconda: Sustainable and Comprehensive Software Distribution for the Life Sciences. Nat. Methods.

[52] Varadi M., Bertoni D., Magana P., Paramval U., Pidruchna I., Radhakrishnan M., Tsenkov M., Nair S., Mirdita M., Yeo J. (2024). AlphaFold Protein Structure Database in 2024: Providing Structure Coverage for over 214 Million Protein Sequences. Nucleic Acids Res..

[53] Jumper J., Evans R., Pritzel A., Green T., Figurnov M., Ronneberger O., Tunyasuvunakool K., Bates R., Žídek A., Potapenko A. (2021). Highly Accurate Protein Structure Prediction with AlphaFold. Nature.

[54] Kim S., Chen J., Cheng T., Gindulyte A., He J., He S., Li Q., Shoemaker B.A., Thiessen P.A., Yu B. (2025). PubChem 2025 Update. Nucleic Acids Res..

[55] Hastings J., Owen G., Dekker A., Ennis M., Kale N., Muthukrishnan V., Turner S., Swainston N., Mendes P., Steinbeck C. (2016). ChEBI in 2016: Improved Services and an Expanding Collection of Metabolites. Nucleic Acids Res..

[56] Eastman P., Galvelis R., Peláez R.P., Abreu C.R.A., Farr S.E., Gallicchio E., Gorenko A., Henry M.M., Hu F., Huang J. (2024). OpenMM 8: Molecular Dynamics Simulation with Machine Learning Potentials. J. Phys. Chem. B.

[57] Landrum G., Tosco P., Kelley B., Rodriguez R., Cosgrove D., Vianello R., sriniker, Gedeck P., Jones G., Kawashima E. Rdkit/Rdkit: 2025_03_5 (Q1 2025) Release 2025. https://ui.adsabs.harvard.edu/abs/2025zndo..17232453L/abstract.

[58] Eberhardt J., Santos-Martins D., Tillack A.F., Forli S. (2021). AutoDock Vina 1.2.0: New Docking Methods, Expanded Force Field, and Python Bindings. J. Chem. Inf. Model..

[59] Trott O., Olson A.J. (2010). AutoDock Vina: Improving the Speed and Accuracy of Docking with a New Scoring Function, Efficient Optimization, and Multithreading. J. Comput. Chem..

[60] Pettersen E.F., Goddard T.D., Huang C.C., Couch G.S., Greenblatt D.M., Meng E.C., Ferrin T.E. (2004). UCSF Chimera—A Visualization System for Exploratory Research and Analysis. J. Comput. Chem..

[61] Schake P., Bolz S.N., Linnemann K., Schroeder M. (2025). PLIP 2025: Introducing Protein–Protein Interactions to the Protein–Ligand Interaction Profiler. Nucleic Acids Res..

