# Supplementary material for: Unveiling Specificity, Redundancy, and Promiscuity of Five Saccharomyces cerevisiae Mitochondrial Carriers
Source: Int J Mol Sci. 2026 Jan 31;27(3):1450. doi: 10.3390/ijms27031450 (PMC12897880; doi:10.3390/ijms27031450)
Supplement: Supplementary file 1 [file ijms-27-01450-s001.zip › ijms-4113875-supplementary.pdf]

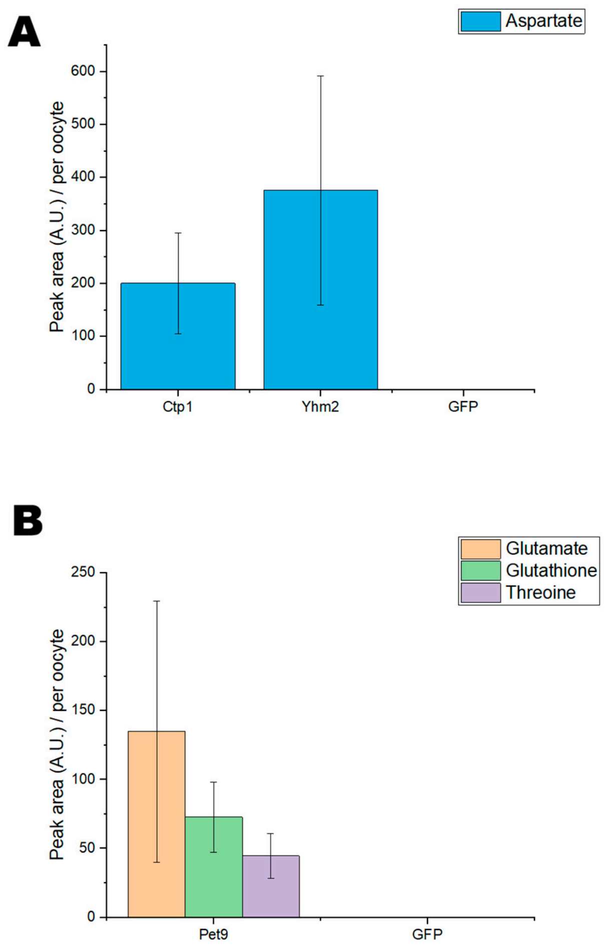

Supplementary Figure S1. Uptake of individual amino acids in *X. laevis* oocyte producing single mitochondrial transporters. (A) Uptake of aspartate by Xenopus oocytes producing MC Ctp1, Yhm2 or cytosolic GFP as control. (B) Uptake of glutamate, glutathione and threonine by Xenopus oocytes producing Pet9 or cytosolic GFP as control. Between four and six oocytes were utilized per replicate and between three and four replicates were assayed against the OAM. Error bars indicate standard deviation.

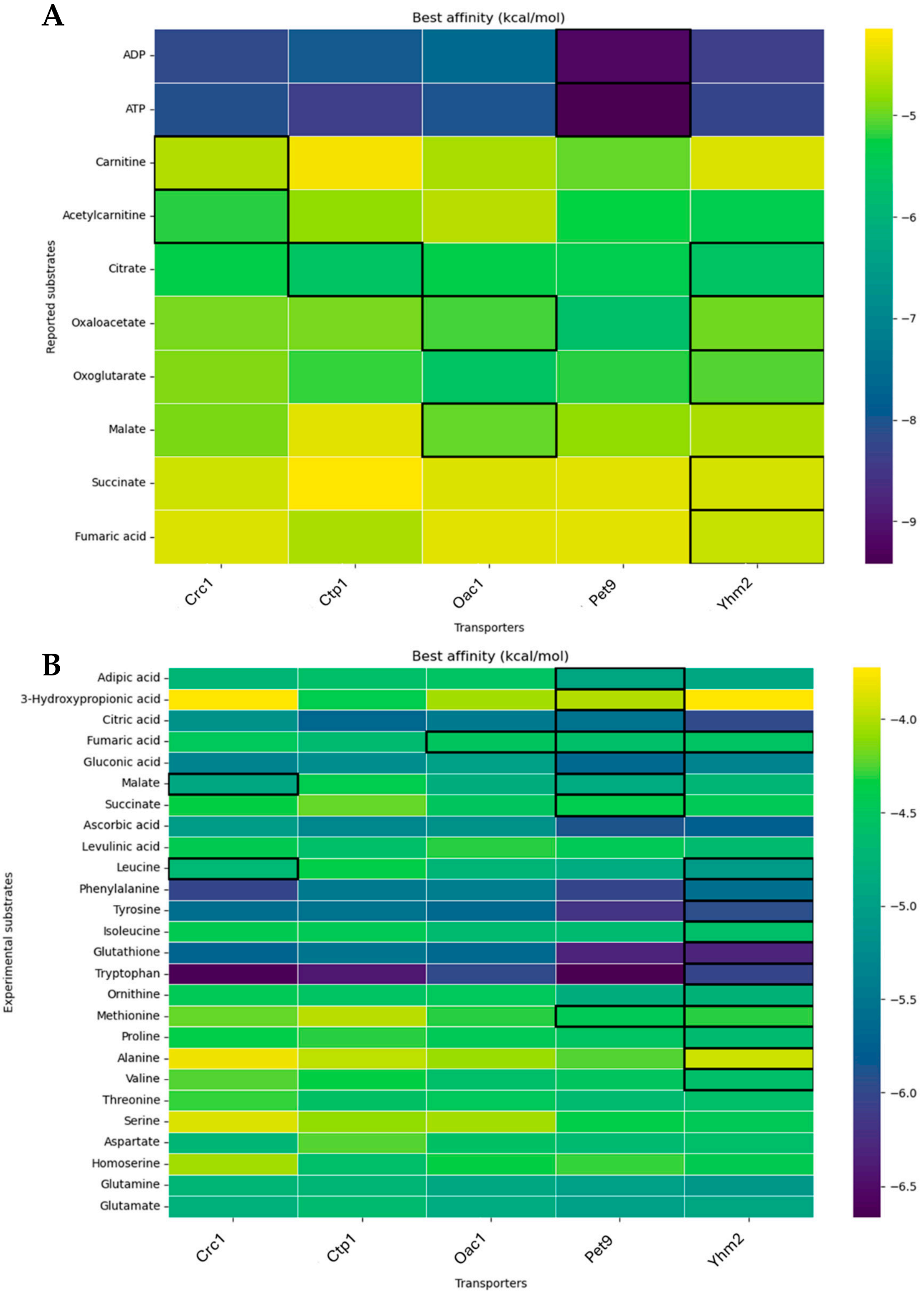

Supplementary Figure S2. Docking results for mitochondrial transporters. (**A**) Docking results for reported substrates. Heatmap showing the best binding affinities (lowest docking energy in kcal/mol) for each transporter-substrate pair. Black boxes indicate substrates reported to be transported by the corresponding protein. Malate, succinate, and fumaric acid were included despite being minor or secondary substrates, as they were part of the experimental transport assay (Supplementary Table 1) and have been previously reported. (B) Heatmap showing the best binding affinities (lowest free docking energy in kcal/mol) for each transporter-substrate pair. Black boxes indicate substrates that

were found to be significantly transported by the corresponding protein in the experimental assay with p values <0.05.

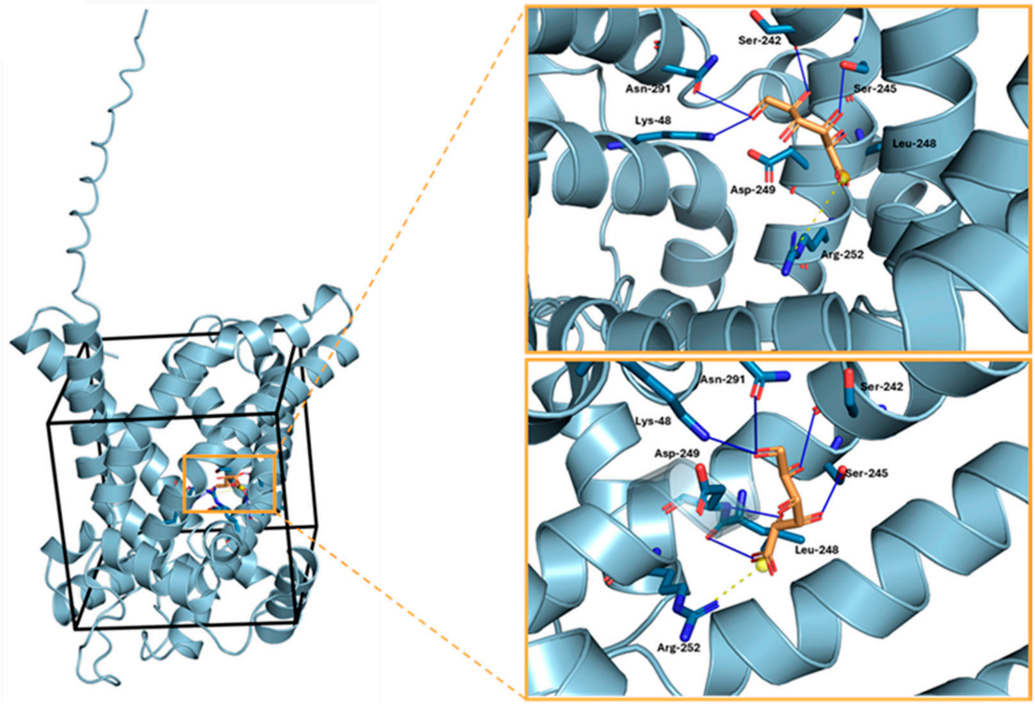

Supplementary Figure S3. **Predicted binding pose of Gluconic acid in Pet9 from molecular docking.** Left: Overall structure of Pet9 rendered in cartoon representation (PyMOL), with the predicted substrate binding site boxed in orange. The black box represents the grid box (30x30x30 Å) used in the docking simulations to define the search space. Right: Two magnified views of the binding site from different orientations, showing the docked substrate and interacting residues. Gluconic acid is shown in orange while side chains of interacting residues are shown as blue sticks for nitrogen and red sticks for oxygen. Key interactions are illustrated. The substrate forms seven hydrogen bonds (dark blue) and one salt bridge (yellow) with residues at the binding site.

Supplementary Table S1: Organic acid mix used for uptake assays. Concentration of all organic acids is 500 μM.

**Compound Name**	**Abbreviation**	**Chemical Formula**	**Molecular Weight (g/mol)**
Acetic acid	Ace	$C2H4O2$	60.05
Oxalic acid	Oxa	$C2H2O4$	90.03
Acrylic acid	Acr	$C3H4O2$	72.06
3-hydroxypropionic acid	3HP	$C3H6O3$	90.08
Lactic acid	Lac	$C3H6O3$	90.08
Propionic acid	Pro	$C3H6O2$	74.08
Butyric acid	But	$C4H8O2$	88.11
Fumaric acid	Fum	$C4H4O4$	116.07
Malic acid	Mal	$C4H6O5$	134.09
Succinic acid	Suc	$C4H6O4$	118.09
Itaconic acid	Ita	$C5H6O4$	130.10
Levulinic acid	Lev	$C5H8O3$	116.11
Adipic acid	Adi	$C6H10O4$	146.14
Ascorbic acid	Asc	$C6H8O6$	176.12
Citric acid	Cit	$C6H8O7$	192.12
Gluconic acid	GluA	$C6H12O7$	196.16
a-ketoglutaric acid	aKG	$CH6O5$	146.11

Supplementary Table S2. Oligonucleotide primers used in this study.

Name	Sequence (5′ to 3′)	Orientation	Description
PR3386	GGCTTAAUATGTCCAGTAAAGCTACCAAAAGT	→	Amplification of CTP1 from gDNA
PR33866	GGTTTAAUTCAGGCTAGCATAACTAAGACCTTTTC	←	
PR33951	GGCTTAAUATGTCTTCAGACACTTCATTATCA	→	Amplification of CRC1 from gDNA
PR33952	GGT TTAATCATATGCCATACTTCTTGAACA	←	
PR33889	GGCTTAAATGTCATCTGACAACTCTAAACAA	→	Amplification of OAC1 from gDNA
PR33890	GGTTTAAUTTAATTATGGCCTAAAACTCTCGA	←	
PR33859	GGCTTAAUATGT CTTCCAACGCCCAA	→	Amplification of PET9 from gDNA
PR33860	GGTTTAAUTTATTTGAACTTCTTACCAAACAAGATC	←	
PR33881	GGCTTAAATGGCACTACGATTTTTCAAC	→	Amplification of POR2 from gDNA
PR33882	GGTTTAATCAGGGCGAGAACGATAG	←	
PR33931	GGCTTAAUATGCCATCTACCACTAATACTG	→	Amplification of YHM2 from gDNA
PR33932	GGTTTAAUCTAATGTTTGGCAACTGGGG	←	

Supplementary Table S3. Plasmids used in this study.

Name	Description	Reference
pCfB5245	USER-compatible *Xenopus gene* expression vector	[35]
pCfB13021	*Xenopus* gene expression vector for CTP1	This study
pCfB13033	*Xenopus* gene expression vector for CRC1	This study
pCfB13032	*Xenopus gene* expression vector for OAC1	This study
pCfB13031	*Xenopus gene* expression vector for PET9	This study
pCfB13026	*Xenopus gene* expression vector for POR2	This study
pCfB13030	*Xenopus* gene expression vector for YHM2	This study

Supplementary Table S4. Raw data from docking simulations.

Name	Binding affinities of top scoring poses (kcal/mol)				
	Crc1	Ctp1	Oac1	Pet9	Yhm2
Adipic acid	-4.707	-4.575	-4.534	-4.933	-4.903
3-Hydroxypropionic acid	-3,733	-4,387	-4,039	-3,989	-3,725
Citric acid	-5.163	-5.686	-5.457	-5.527	-5.970
Fumaric acid	-4.474	-4.671	-4.517	-4.587	-4.547
Gluconic acid	-5.313	-5.205	-4.986	-5.634	-5.352
Malate	-4.909	-4.390	-4.823	-4.873	-4.690
Succinate	-4.335	-4.197	-4.495	-4.395	-4.434
Ascorbic acid	-5.044	-5.300	-5.183	-5.871	-5.774
Levulinic acid	-4.416	-4.577	-4.318	-4.444	-4.622
Leucine	-4.678	-4.351	-4.751	-4.835	-5.047
Phenylalanine	-6.002	-5.449	-5.419	-6.001	-5.555
Tyrosine	-5.575	-5.548	-5.652	-6.185	-5.925
Isoleucine	-4.420	-4.431	-4.655	-4.676	-4.601
Glutathione	-5.689	-5.493	-5.655	-6.333	-6.298
Tryptophan	-6.638	-6.416	-5.984	-6.663	-6.042
Ornithine	-4.433	-4.506	-4.477	-4.808	-4.762
Methionine	-4.199	-3.984	-4.320	-4.428	-4.302
Proline	-4.364	-4.306	-4.451	-4.516	-4.651
Alanine	-3.799	-3.958	-4.069	-4.247	-3.924
Valine	-4.243	-4.337	-4.603	-4.505	-4.587
Threonine	-4.293	-4.570	-4.481	-4.675	-4.601
Serine	-3.871	-4.082	-4.039	-4.367	-4.462
Aspartate	-4.699	-4.236	-4.556	-4.676	-4.611
Homoserine	-4.042	-4.609	-4.343	-4.278	-4.425
Glutamine	-4.737	-4.715	-4.905	-5.002	-5.105
Glutamate	-4.803	-4.648	-4.858	-5.003	-4.933
ADP	-7.718	-7.907	-7.425	-9.121	-8.354
ATP	-8.078	-8.220	-8.242	-9.793	-8.805
Carnitine	-4.597	-4.224	-4.668	-5.005	-4.531
Acetylcarnitine	-5.124	-4.786	-4.604	-5.238	-4.642
Citrate	-5.270	-5.547	-5.299	-5.449	-5.869
Oxaloacetate	-4.963	-4.952	-5.160	-5.697	-5.011
Oxoglutarate	-4.890	-5.175	-5.502	-5.262	-5.069
Malate	-4.909	-4.390	-4.823	-4.873	-4.690
Succinate	-4.335	-4.197	-4.495	-4.395	-4.434

Supplementary Table S5. Dimensions of grid boxes used to define docking search space.

Transporter	Box Center (Å)	Box Size (Å)
Crc1	(0, 0, 0)	(30, 30, 30)
Ctp1	(3, 0, -2)	(30, 30, 30)
Oac1	(-5, 0, -3)	(30, 30, 30)
Pet9	(0, 0, 0)	(30, 30, 30)
Yhm2	(-5, 0, 3)	(30, 30, 30)
